# A single-centred retrospective observational analysis on mortality trends during the COVID-19 pandemic

**DOI:** 10.4102/safp.v65i1.5700

**Published:** 2023-06-05

**Authors:** Omishka Hirachund, Camilla Pennefather, Mergan Naidoo

**Affiliations:** 1 Discipline of Family Medicine, School of Nursing and Public Health, University of KwaZulu-Natal, Durban, South Africa

**Keywords:** severe acute respiratory syndrome coronavirus 2, SARS-CoV-2, COVID-19, mortality trends, risk factors for mortality, district hospital, South Africa

## Abstract

**Background:**

South Africa experienced high mortality during the COVID-19 pandemic. Resources were limited, particularly at the district hospital (DH) level. Overwhelmed healthcare facilities and a lack of research at a primary care level made the management of patients with COVID-19 challenging. The objective of this study was to describe the in-hospital mortality trends among individuals with COVID-19 at a DH in South Africa.

**Methods:**

Retrospective observational analysis of all adults who demised in hospital from COVID-19 between 01 March 2020 and 31 August 2021 at a DH in South Africa. Variables analysed included: background history, clinical presentation, investigations and management.

**Results:**

Of the 328 participants who demised in hospital, 60.1% were female, 66.5% were older than 60 and 59.6% were of black African descent. Hypertension and diabetes mellitus were the most common comorbidities (61.3% and 47.6%, respectively). The most common symptoms were dyspnoea (83.8%) and cough (70.1%). ‘Ground-glass’ features on admission chest X-rays were visible in 90.0% of participants, and 82.8% had arterial oxygen saturations less than 95% on admission. Renal impairment was the most common complication present on admission (63.7%). The median duration of admission before death was four days (interquartile range [IQR]: 1.5–8). The overall crude fatality rate was 15.3%, with the highest crude fatality rate found in wave two (33.0%).

**Conclusion:**

Older participants with uncontrolled comorbidities were most likely to demise from COVID-19. Wave two (characterised by the ‘Beta’ variant) had the highest mortality rate.

**Contribution:**

This study provides insight into the risk factors associated with death in a resource-constrained environment.

## Introduction

Severe acute respiratory syndrome coronavirus 2 (SARS-CoV-2) is responsible for the COVID-19 pandemic. Clinical presentation of the illness ranges from mild, flu-like symptoms, moderate symptoms to severe, pneumonia-like symptoms.^1^ During the first three waves and prior to the vaccination rollout, state-run and private healthcare facilities across the country were overwhelmed by the impact of COVID-19.^2^ In a South African district hospital (DH) setting, ventilators, high-flow nasal oxygen (HFNO2) devices and non-invasive continuous positive airway pressure (CPAP) machines were scarce when compared to that in high-income countries (HICs).^2,3^

The South African public healthcare system consists of different levels of hospitals, namely district, regional, tertiary, provincial tertiary, central and specialised. District hospitals provide primary healthcare and unspecialised services and subsequently refer patients to regional or tertiary facilities should the patient require a higher level of care. All patients presenting to DHs are primary presentations or referrals from primary care clinics. Screening for COVID-19 is conducted at the entrance of the clinic or hospital. Patients are evaluated by a clinician and, if COVID-19 antigen or polymerase chain reaction (PCR) positive and deemed necessary, are admitted to the ‘Person-Under-Investigation (PUI)’ ward. Patients are subsequently transferred to the general COVID-19 ward (if clinically stable) or to the ‘high-care’ COVID-19 ward (if requiring a higher level of care or invasive ventilation). Those with a negative COVID-19 antigen or PCR test are transferred to the non-COVID-19 general ward or ‘high care’. A locally devised probability scoring tool (defined below) was used when antigen testing was not yet introduced in South Africa, and clinicians could not delay the focussed treatment of patients for the 10–14 days that it took for the PCR result to be ready.

During the peak of each wave, many tertiary, regional and central hospitals were running at maximum capacity, and intensive care unit (ICU) services were limited, resulting in poor acceptance rates or long waiting times for referrals from DHs. Consequently, many severely ill and complex patients were managed at primary care DHs. Under-resourced healthcare facilities, pandemic fatigue (among healthcare workers and other support staff), a lack of research at a district healthcare level and the quadruple burden of disease made the management of patients with COVID-19 and subsequent preparation for future waves or pandemics challenging – particularly at a DH level. Numerous studies regarding risk factors for mortality have been published internationally, and much is known about the socio-economical and health-related consequences of the pandemic on various countries worldwide.^4,5^ There is limited mortality data from primary care facilities in both low- and middle-income countries (LMICs), particularly from DHs in a South African setting.^2,4^

The objective of the following study was to describe the in-hospital mortality trends among individuals with COVID-19 at Wentworth, a DH in South Africa. Determining these risk factors will assist with identifying high-risk populations, optimising future healthcare planning and preparing for subsequent waves or disease outbreaks. Additionally, it will allow healthcare providers to reflect on the impact of COVID-19 at a primary care level.

## Methods

The study was a retrospective descriptive analysis of clinical records from Wentworth Hospital (WWH). Wentworth Hospital is a DH situated in the Wentworth district of Durban, South Africa. The target population included all adult participants over 18 years who were admitted to WWH and demised in hospital from COVID-19 disease between 01 March 2020 and 31 August 2021. During this period, South Africa experienced three waves (periods of increased transmission) as defined by the National Institute of Communicable Disease (NICD)^6^: wave 1 in KwaZulu-Natal is defined as week 26–34 (2020), wave 2 is defined as week 49–5 (2020–2021) and wave 3 is defined as week 24–37 (2021). The institutional crude fatality rate (institutional COVID-19 deaths/institutional COVID-19 admissions) for the entire study period, as well as for the defined waves of infection, was calculated.

The diagnosis of COVID-19 disease was based on clinical, laboratory or radiological features suggestive of SARS-CoV-2 infection. The PCR test was used to confirm the diagnosis of COVID-19, and only participants who demised in hospital because of COVID-19 were included in the study. Because of the long turnaround time for the COVID-19 PCR test (up to 10 days), a locally devised clinical scoring system was used to identify participants who were strongly suspicious of having COVID-19 disease. A score of 9 or greater (out of a total of 21) was deemed to be highly suggestive of COVID-19. The PCR test later confirmed these results or, if the PCR test was negative, clinical judgement, as well as biochemical and radiological markers were used to conclude the diagnosis of COVID-19 using the probability score. The scoring system comprised the following criteria: a history of positive contact (2 points), acute cough (1 point), fever > 38 °C (2 points), respiratory rate > 25/min (1 point), pulse oximetry saturation (SpO_2_) < 95% (2 points), recent loss of taste taste or smell (3 points), high C-reactive protein (CRP) (2 points), high white cell count (WCC) (1 point), positive chest X-ray (ground glass [3 points] or pneumonia [2 points]), D-dimer > 0.25 (2 points) and any diabetic emergency (hyper or hypoglycaemia) (2 points). This was an institutionally devised scoring system by two South African family physicians and an external international critical care specialist whereby a score of nine or more was highly indicative of COVID-19. The tool was not validated or used elsewhere.

An electronic data collection tool was devised for the study. It was initially developed by one of the authors and was used as a mortality audit tool. Data were captured through a Google Form and presented at the monthly audit meetings. However, it required additional information to be added to the original tool as it was not comprehensive enough to answer the research objectives. Data from paper-based patient records were captured on a spreadsheet by the two principal investigators and analysed. The editor then checked the data. All the study objectives could be achieved with the modified data extraction tool and was subsequently used for data capture. The pilot study – which was conducted on the files of 54 participants who demised in August 2021 – allowed further adjustment of the data collection tool to extract meaningful, non-ambiguous data. The data collection tool included variables such as demographics, comorbidities, clinical presentation, biochemical findings, radiological markers and management. Race was captured from the COVID-19 admission clerking tool, as self-identified by participants. For those who were dead on arrival or too ill to communicate, race was reported by the next of kin of the deceased. The chest X-ray done on admission was evaluated by the admitting doctor and confirmed by the family physician in charge (as there was no radiologist available for reporting). This was documented on the COVID-19 admission clerking tool. Peripheral pulmonary infiltrates were classified as ‘mild’ changes, and ‘ground glass appearance’ or ‘global consolidation’ was classified as ‘moderate/severe’ changes.

Descriptive statistics were used to summarise the data. Frequencies and percentages were used for categorical data such as gender, ethnicity and comorbidities. Frequency distributions of numeric variables were examined for normality and mean (standard deviation [s.d.]) or median (interquartile range [IQR]) were used when appropriate. Stata^®^ V15.1^7^ was used for the analysis.

## Results

### Baseline characteristics

Of the 328 participants who demised from COVID-19, 197 (60.1%) were female (Table 1). The mean age of death was 64 years (standard deviation [s.d.]: 13.6), with the most significant proportion of deaths (*n* = 218; 66.5%) occurring in individuals older than 60 years. Most participants (*n* = 196; 59.6%) were of black African descent. Of those with documented clinical frailty scores (*n* = 312), 197 (63.1%) had a score of less than six, indicating good pre-morbid function. Hypertension was present in 201 (61.3%) participants and diabetes mellitus in 156 (47.6%) participants, with the majority (*n* = 115; 73.7%) of these having an haemoglobin A1c (HbA1c) greater than 8%. Of the total study population, 62 (18.9%) participants had a documented weight, all of whom had a body mass index (BMI) greater than 30, classifying them as ‘obese’. Fifty-three (16.2%) participants had documented dyslipidaemia. Forty-five (13.7%) participants were human immunodeficiency virus (HIV)-positive, with 37 (82.2%) having unsuppressed/unknown viral loads. Most (*n* = 237; 72.3%) participants reported sober habits.

**TABLE 1:** Demographics and relevant clinical history of participants.

Variable (*n* = 328)	Number (*n*)	%	Mean	s.d.
**Baseline characteristics**				
Female	197	60.1	-	
Age (years)	-	-	63.5	13.6
> 60 years	218	66.5	-	-
< 60 years	110	33.5	-	-
**Ethnic classification**				
Black person	196	59.6	-	-
White person	50	15.2	-	-
Asian person	48	14.6	-	-
Mixed-race person	34	10.4	-	-
**Clinical frailty score (*n* = 312)†**				
≤ 5	197	63.1	-	-
> 5	115	36.9	-	-
**Comorbidities**				
Hypertension	201	61.3	-	-
Diabetes mellitus	156	47.6	-	-
Of which HbA1c less than 7% (*n* = 156)	8	5.1	-	-
Of which HbA1c 7-8 (*n* = 156)	33	21.2	-	-
Of which HbA1c greater than 8% (*n* = 156)	115	73.7	-	-
Obese (body mass index greater than 30)	62	18.9	-	-
Dyslipidaemia	53	16.2	-	-
HIV-positive	45	13.7	-	-
Of which viral load unsuppressed/unknown (*n* = 45)	37	82.2	-	-
Current tuberculosis	10	3.0	-	-
Previous tuberculosis	13	4.0	-	-
Asthma	11	3.4	-	-
COPD	8	2.4	-	-
Chronic kidney disease	19	5.8	-	-
Ischaemic heart disease	31	9.5	-	-
Malignancy	9	2.7	-	-
**Habits**				
Sober habits	237	72.3	-	-
Current smoking	40	12.2	-	-
Ex-smoker	16	4.9	-	-
Current alcohol use	17	5.2	-	-
Illicit drug use	2	0.6	-	-

s.d., standard deviation; HbA1c, glycated haemoglobin; COPD, chronic obstructive pulmonary disease.

† , Except where specified.

### Clinical presentation

The most frequent presenting symptoms were dyspnoea (83.8%) and cough (70.1%) (Table 2). Modified Medical Research Council (mMRC) dyspnoea grade 4 was evident in 143 (43.6%) participants on admission. Most participants (57.6%) were tachypnoeic (respiratory rate greater than 25 breaths per minute), and 55.8% were tachycardic (heart rate ≥ 100 beats per minute). The mean systolic blood pressure was 128.7 (s.d.: 26.4), and the mean diastolic blood pressure was 74.2 (s.d.: 18.2) mmHg. The mean SpO_2_ on room air was 77.2% (s.d.: 18.8), with 82.8% of participants having an SpO_2_ < 95% on admission. The median finger-prick blood glucose on admission was 8.3 mmol/L (IQR: 6.6–15.6), and 206 (62.8%) participants had a blood glucose ≤ 11 mmol/L. On presentation to the emergency department, 235 (71.6%) participants had a Glasgow Coma Score (GCS) of 15, 69 (21.0%) had a GCS between 10 and 14, 16 (4.9%) had a GCS between three and nine and eight (2.4%) were unspecified. The majority (68.8%) of participants were triaged as an ‘orange-code’ on admission as defined by the South African Triage Scale (SATS), and a clinical probability score of nine or more was recorded in 275 (83.8%) participants.

**TABLE 2:** Clinical presentation on admission of participants.

Variable (*n* = 328)†	Number (*n*)	%	Mean	s.d.	Median	IQR
**Symptoms**						
Shortness of breath	278	83.8	-	-	-	-
Cough	230	70.1	-	-	-	-
Myalgia/body pain	128	39.0	-	-	-	-
Fever	96	29.3	-	-	-	-
Sore throat	59	18.0	-	-	-	-
Confusion	59	18.0	-	-	-	-
Recent loss of taste or smell	43	13.1	-	-	-	-
Diarrhoea	40	12.2	-	-	-	-
Chest pain	24	7.3	-	-	-	-
Headache	13	4.0	-	-	-	-
Other (unspecified)	45	13.7	-	-	-	-
mMRC grade 4 dyspnoea	143	43.6	-	-	-	-
**Vital signs**						
Respiratory rate	-	-	27.7	8.3	-	-
≥ 25 breaths per minute	189	57.6	-	-	-	-
< 25 breaths per minute	132	40.2	-	-	-	-
Heart rate	-	-	103	21.7	-	-
≥ 100 beats per minute	183	55.8	-	-	-	-
< 100 beats per minute	137	41.8	-	-	-	-
Systolic blood pressure	-	-	128.7	26.4	-	-
Diastolic blood pressure	-	-	74.2	18.2	-	-
SP0_2_ on room air (*n* = 319)	-	-	77.2	18.8	-	-
< 95	264	82.8	-	-	-	-
> 95	55	17.2	-	-	-	-
**Glasgow Coma Score**						
15	235	71.6	-	-	-	-
10–14	69	21.0	-	-	-	-
3–9	16	4.9	-	-	-	-
Unspecified	8	2.4	-	-	-	-
**Finger-prick blood glucose**	-	-	-	-	8.3	6.6–15.6
≤ 11 mmol/L	206	62.8	-	-	-	-
> 11 mmol/L	115	35.1	-	-	-	-
**Triage colour on admission**						
Red	55	16.8	-	-	-	-
Orange	225	68.8	-	-	-	-
Yellow	36	11	-	-	-	-
Green	11	3.4	-	-	-	-
**Clinical probability score on admission**	-	-	10.76	2.49	-	-
Less than nine	48	14.6	-	-	-	-
Nine or more	275	83.8	-	-	-	-
Unspecified	-	1.5	-	-	-	-

mMRC, Modified Medical Research Council; s.d., standard deviation; IQR, interquartile range.

† , Except where specified.

### Investigations

Of all the participants included in the study, 311 (94.8%) had a positive PCR test and 172 (76.1%) had a positive rapid antigen test (Table 3). A total of 329 (97.9%) participants were unvaccinated at the time of death. No participants were fully vaccinated at the time of death. Moderate/severe chest radiograph changes were evident in 278 (90.0%) participants. The median Horowitz Index^8^ (ratio of arterial partial pressure of oxygen to fraction of inspired oxygen the patient is receiving: PaO_2_/FiO_2_) was 125 (IQR: 75–250), with 40.9% of participants having a Horowitz Index^9^ less than or equal to 100, indicating severe acute respiratory distress syndrome (ARDS) on presentation. Biochemical derangements included a median urea of 9.4 (IQR: 5.9–17.1), median creatinine of 112 (IQR: 80–208) and a median glomerular filtration rate (GFR) of 47 (IQR: 25–82). The median CRP was 150.8 (s.d.: 65.94), median D-dimer was 0.69 (IQR: 0.33–1.18) and mean lactate dehydrogenase (LDH) was 1413.53 (s.d.: 621.56).

**TABLE 3:** Investigations of participants.

Variable (*n* = 328)	Number (*n*)	%	Mean	s.d.	Median	IQR
**Investigations**						
**COVID-19 data**	-	-	-		-	-
Positive PCR	311	94.8	-		-	-
Positive antigen (*n* = 226)	172	76.1	-		-	-
Vaccinated fully	0	0	-		-	-
Partially vaccinated (one dose Pfizer)	2	0.6	-		-	-
Unvaccinated	321	97.9	-		-	-
Unknown vaccination status	5	-	-		-	-
**Chest X-ray findings**						
Changes on chest X-ray documented	309	94.2	-	-	-	-
Of which mild changes (*n* = 309)	31	10.0	-	-	-	-
Of which moderate to severe changes (*n* = 309)	278	90.0	-	-	-	-
**Horowitz Index9 on admission (*n* = 254)**	-	-	-	-	125	75–250
≤ 100	104	40.9	-		-	-
101–200	64	25.2	-		-	-
200–300	86	33.9	-		-	-
**Urine dipstick**						
Normal	5	1.5	-	-	-	-
Glucose	58	17.7	-	-	-	-
Protein	58	17.7	-	-	-	-
Ketones	26	7.9	-	-	-	-
Blood	19	5.8	-	-	-	-
Leukocytes	10	3.0	-	-	-	-
Not done/unspecified	220	67.1	-	-	-	-
**Blood results**						
Haemoglobin (*n* = 311)	-	-	12.45	2.49	-	-
White cell count (*n* = 311)	-	-	11.24	5.61	-	-
Platelets (*n* = 310)†	-	-	-	-	273	211; 357
Urea (*n* = 314)	-	-	-	-	9.4	5.9–17.1
Creatinine (*n* = 313)	-	-	-	-	112	80–208
GFR (*n* = 311)	-	-	-	-	47	25–82
≥ 60	123	39.5	-	-	-	-
< 60	188	60.5	-	-	-	-
Bilirubin (*n* = 290)	-	-	-	-	12	9–19
ALT (*n* = 288)	-	-	-	-	28	20–47
GGT (*n* = 286)	-	-	-	-	43	24–85
CRP (*n* = 310)	-	-	150.8	65.94	-	-
≥ 200	144	46.5	-	-	-	-
< 200	166	53.5	-	-	-	-
D-dimer (*n* = 278)	-	-	-	-	0.69	0.33–1.18
≥ 0.5	174	62.6	-	-	-	-
< 0.5	104	37.4	-	-	-	-
LDH (*n* = 40)	-	-	1413.53	621.56	-	-

PCR, polymerase chain reaction; Horowitz Index^9^, ratio of arterial partial pressure of oxygen to fraction of inspired oxygen the patient is receiving (PaO_2_ / FiO_2_); IQR, interquartile range; s.d., Standard deviation; GFR, glomerular filtration rate; ALT, alanine transaminase; GGT, gamma-glutamyl transferase; CRP, C-reactive protein; LDH, lactate dehydrogenase.

† , Except where specified.

### Complications

Complications noted on admission included renal impairment (63.7%), depressed level of consciousness (22.3%), haemodynamic instability (21.6%), liver enzyme abnormalities (18.6%) and thrombo-embolic disease (10.7%) (Table 4).

**TABLE 4:** Complications and management of participants.

Variable (*n* = 328)	Number (*n*)	%	Median	IQR
**Complications present on admission**				
Renal impairment	209	63.7	-	-
Depressed level of consciousness	73	22.3	-	-
Haemodynamic instability	71	21.6	-	-
Liver enzyme abnormality	61	18.6	-	-
Thrombo-embolic disease	35	10.7	-	-
Diabetic keto-acidosis	29	8.8	-	-
Myocarditis	19	5.8	-	-
Other	12	3.7	-	-
No complication/unspecified	78	23.8	-	-
**Management**				
Treatment prescribed				
Dexamethasone	297	90.5	-	-
LMWH prophylaxis	240	73.2	-	-
LMWH therapeutic	60	18.3	-	-
Thiamine	194	59.1	-	-
Vitamin D stat doses	147	44.8	-	-
Vitamin B3	178	54.3	-	-
Zinc	195	59.5	-	-
Ceftriaxone	304	92.7	-	-
Azithromycin	287	87.5	-	-
**Oxygen requirements on admission (*n* = 329)**				
Room air	74	22.5	-	-
Nasal prongs	8	2.4	-	-
40% Venturi face mask	66	20.1	-	-
100% non-rebreather mask	114	34.7	-	-
Dual oxygen	32	9.7	-	-
High-flow nasal oxygen	22	6.7	-	-
Continuous positive airway pressure	12	3.6	-	-
Intubation and mechanical ventilation	1	0.3	-	-
Number of days on oxygen (*n* = 318)	-	-	3	1–7
0	22	7	-	-
1–6	210	66.0	-	-
≥ 7 days	86	27.0	-	-
**Patient discussed with higher levels of care during admission**				
No	229	72.2	-	-
Yes	88	27.8	-	-
Unknown	11	3.5	-	-
**Number of referrals accepted (*n* = 88)**				
No	73	86.9	-	-
Yes	11	13.1	-	-
Unknown	4	-	-	-

LMWH, low molecular weight heparin; IQR, interquartile range.

† , Except where specified.

### Management

With respect to medical management: 297 (90.5%), participants received Dexamethasone 6 mg intravenously (IVI); 240 (73.2%) received prophylactic low molecular weight heparin (LMWH) (40 mg daily subcutaneously); 60 (18.3%) received therapeutic LMWH (1.5 mg per kilogram daily); 194 (59.1%) received thiamine; 147 (44.8%) received vitamin D stat doses; 178 (54.3%) received vitamin B3; 195 (59.5%) received zinc; 304 (92.7%) received ceftriaxone and 287 (87.5%) received azithromycin (Table 4).

Most (*n* = 255; 77.5%) participants were oxygen requiring on admission, ranging from the nasal cannula to invasive ventilation, with 210 (66%) participants requiring oxygen for 1–6 days, and the median number of days for which oxygen was required being 3 (IQR: 1–7) (Table 4). Of the 88 participants (27.8%) discussed with higher levels of care, 73 (86.9%) were not accepted. Reasons for rejection included: ‘no capacity at higher level of care’ (referring to no bed space or ventilators), ‘patient does not meet criteria for referral’ (referring to poor indication for referral or does not meet other specific ICU criteria) or ‘poor prognosis’ (alluding to the justice principle where limited resources are reserved for patients with a good/moderate prognosis). The 11 referrals that were accepted demised prior to transfer, as they were either accepted very late or there was a major delay in the ambulance transfer.

### Mortality trends

The median length of admission to WWH before death was 4 days (IQR: 1.5–8). Most participants (*n* = 196; 62.4%) demised after regular working hours (16:00–07:59). Eleven participants (3.4%) were dead on arrival (DOA), and 29 (8.8%) demised in the emergency centre (EC) within a few hours of arrival, precluding admission.

**FIGURE 1:**
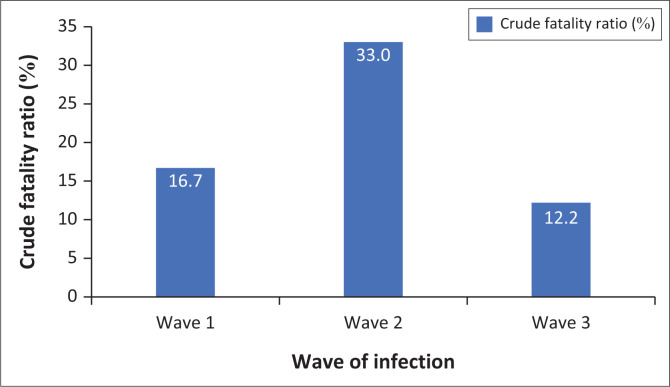
Institutional crude fatality ratios during the first three COVID-19 waves of infection.

A total of 2138 were admitted to WWH from 01 March 2020 to 31 August 2021. During this period, 328 participants demised from COVID-19, yielding an institutional crude fatality rate of 15.3%. Of the total sample size (328), 311 deaths fell into the first three waves of the COVID-19 pandemic as described by the NICD. Fifty-nine (19.0%) of these fell into wave one, 189 (60.8%) fell into wave two and 63 (20.3%) fell into wave three. A total of 354, 573 and 517 patients were admitted to WWH with COVID-19 infection during wave one, two and three, respectively. The crude fatality ratio was therefore 16.7%, 33.0% and 12.2% for waves one, two and three, respectively (Figure 1).

## Discussion

This study describes the demographics, as well as the clinical, biochemical and radiological presentation of 328 participants who demised from COVID-19 at WWH – a single DH – as well as their management. COVID-19 mortality was most frequent in the following participants: females; those older than 60 years; black Africans; obese individuals and those with comorbidities such as hypertension, diabetes mellitus and poorly controlled HIV. On admission, 63.1% participants had clinical frailty scores less than and/or equal to five, indicating good pre-morbid functioning. This highlights the isolated effect of COVID-19 on the immune system and suggests other pathogenic mechanisms for the development of severe infection and death.

The increased mortality rate in elderly individuals is a consistent finding across the published literature.^2,4,10^ Suggested pathogenic mechanisms for this include age-related changes in innate and adaptive immunity and delayed presentation in older individuals because of concerns around exposure to COVID-19 in the hospital environment. An unbalanced, pro-inflammatory environment in elderly individuals can cause exacerbated inflammatory responses, resulting in a cytokine storm in older adults. Such dysregulation of the immune system is the basis of the development of ARDS as well as the involvement of other organ systems leading to multi-organ failure (MOF).^10^

Black African females made up most of the COVID-19-related deaths at WWH. The literature displays a complex interplay between gender, age and race and its associations with COVID-19-related death. A study conducted in the Eastern Cape of South Africa established that gender associations differed with race and age.^9^ Indian males had a higher COVID-19-related death rate, while the lowest death rate was found in coloured females. Overall, females had a higher death rate than males in the black African and mixed race populations, whereas males had a higher death rate than females in the Indian and white populations. The race profile of our participants was likely influenced by the demographic profile of the Wentworth community, which falls within the eThekwini Municipality and had a predominantly black African (74%) majority in the 2011 National Census.^11^ Phaswana-Mafuya et al.^12^ showed that white people were more likely to die at older ages compared to black Africans, mixed race people and Indian people. However, comparing each age group, the death rate was consistently highest among black Africans. These heterogeneous findings implicate complex pathophysiology relating to gender and race associations with COVID-19 death. In addition, poor help-seeking behaviour, a delay in seeking medical attention in advanced disease, low detection rates and limited capacity to manage critical cases have also proven to be risk factors for mortality in LMICs.^4^ This is particularly relevant in this study setting: a resource-limited district hospital – managed mainly by junior medical officers – inundated with a sudden surge of critically ill patients with complex disease processes.

Hypertension, diabetes mellitus and immunocompromised status are reported in several studies (including ours) to be the most common comorbidities in severe COVID-19 infection.^3,5^ The association between hypertension and severe COVID-19 illness and death is postulated to be because of myocardial damage and dysfunction.^13^ This is implied by frequent findings of elevated troponin levels and electrocardiographic abnormalities in these patients. A significant proportion of our participants had diabetes mellitus, most of which had poorly controlled disease, as displayed by high HbA1c levels. The postulated reason for severe COVID-19 disease in participants with diabetes mellitus (primarily with poor glycaemic control) is a weakened innate and humoral immunity secondary to chronic hyperglycaemia, resulting in compromised host defence against SARS-CoV-2.^13^ Diabetes mellitus is associated with low-grade chronic inflammation, making the participant vulnerable to an exaggerated inflammatory response, resulting in ARDS and MOF. Recent studies have also shown a direct damaging effect of SARS-CoV-2 on the pancreas, which worsens hyperglycaemia and possibly triggers the onset of diabetes mellitus in diabetes-naïve individuals.^14^ Obesity is documented in several studies to be associated with severe COVID-19 disease.^5,14^ Obesity was under-reported in our study because of poor documentation in participants’ clinical records and the difficulty experienced by healthcare workers in weighing very ill patients. Nonetheless, all the participants in our study with a documented weight were obese. Most of the HIV-positive participants in our study had unsuppressed viral loads. Although not recorded in our study, the possible associated low CD4+ T-cell count in these participants is explained in a systematic review done by Chen et al.,^10^ which found that lymphopenia (consisting of depletion of both CD4+ and CD8+ T-cells) and high neutrophil counts are helpful predictors for COVID-19 death. In contrast, high lymphocyte counts predict better clinical outcomes.

Most of our participants exhibited sober habits; however, the strongest association (albeit weak) was seen with current cigarette smoking. Alqahtani et al.^15^ found that current smokers were 1.45 times more likely to have severe COVID-19-related complications and had a higher mortality rate of 38.5% compared to non-smokers and previous smokers. A study conducted on 4244 patients admitted with severe COVID-19 disease to ICUs across Switzerland, Belgium and France found that only 4% were active smokers.^5^ Van Zyl-Smit et al.^16^ postulated that increased susceptibility to infection in current smokers may be because of upregulation of the angiotensin-converting enzyme 2 (ACE2) receptor, which is the main receptor responsible for the entry of SARS-CoV-2 into the host’s mucosa. It is still unclear as to whether modification of ACE 2 receptor availability affects COVID-19-related mortality.

The most common presenting symptoms were dyspnoea, cough, myalgia, fever, sore throat, confusion, loss of taste and/or smell and diarrhoea. A systematic review by Kumar Ochani et al.^17^ delineated the most common symptoms reported by patients throughout the disease course, including all the symptoms mentioned above. These clinical presentations varied slightly between the different strains of SARS-CoV-2 viruses and different individuals; however, the core symptoms remained the same. Modified Medical Research Council grade four dyspnoea was present in many of our participants, highlighting the propensity for severe COVID-19 to cause ARDS, particularly in older individuals. This symptom is congruent with the chest X-ray changes on admission, most of which were classified as ‘moderate-severe’ with peripheral ground glass opacities – which has been evident in various other studies.^18,19^ Furthermore, most of our participants were tachypnoeic with low arterial oxygen saturations on admission, likely because of ARDS.

On 17 February 2021, South Africa commenced its national vaccine rollout strategy, which was initially only available to healthcare workers.^20^ People aged 60 years and older were only eligible to get the vaccine on 17 May 2021. People aged 50–59 years were only eligible to get the vaccine on 05 July 2021. Booster doses became available on 10 November 2021.^21^ Even though vaccination was unavailable to most of the participants in our cohort, fewer than expected were partially vaccinated (only two participants) and none were fully vaccinated. This perhaps suggests a protective effect of vaccination against COVID-19-related death. Reasons for the initial lag in vaccination programme uptake likely include stigma/fear surrounding the vaccine, a lack of knowledge/health promotion around the topic, as well as a lack of access to vaccination facilities. Despite different dose requirements, efficacies, duration of immunity and side-effect profiles, approved vaccinations reduce the risk of COVID-19-associated deaths.^10^

The introduction of COVID-19 rapid antigen testing in KwaZulu-Natal in December 2020 improved the speed of COVID-19 diagnosis, making it a quick and reliable adjunct to diagnosing COVID-19. A recent Cochrane review on antigen testing stated that in symptomatic patients, ‘some rapid antigen tests are accurate enough to replace RT–PCR, especially for ruling in the presence of infection’.^22^ Despite this, SARS-CoV-2 PCR is still the gold standard for diagnosis of COVID-19 infection with a recent meta-analysis stating a sensitivity of 86% on a nasopharyngeal swab.^18^ Most participants in our study had a positive PCR result (94.8%), with a small proportion of participants included in the study despite a negative PCR result. These participants were included based on locally defined radiological and clinical criteria (high probability score) suggestive of COVID-19. This score allowed for rapid triage of patients with an increased probability of having COVID-19 to be admitted to the isolation wards, as the PCR turnaround time exceeded 10 days during the first two waves. Most participants had raised inflammatory- (CRP, LDH) and pro-thrombotic (D-dimer) markers, in keeping with the proposed pro-inflammatory and pro-thrombotic nature of COVID-19 disease. Chen et al.^10^ explain the microvascular thrombosis and MOF seen in severe COVID-19 disease to be linked to the complement activation and endothelial dysfunction caused by immune and inflammatory markers, contributing to a dysregulation of the coagulation system. This is the basis for prophylactic administration of LMWH and intravenous immune globulin (IVIg) to patients with severe COVID-19.

The most frequent complication noted on admission was an impaired renal function; however, the chronicity of the renal dysfunction was not established. Because the derangements were present from admission, this could be indicative of the typical patient presenting with severe COVID-19 disease: multimorbid patient with established chronic kidney disease. Renal replacement therapy was needed in 28% of a cohort of patients admitted with severe COVID-19 disease to several ICUs in western Europe.^5^ About one quarter of our participants had a decreased level of consciousness and haemodynamic instability on admission, indicating the systemic instability of patients with severe COVID-19 disease and proving useful clinical tools for predicting poor clinical outcomes and mortality. Thrombo-embolic complications were also common in patients with severe COVID-19 disease, as evident by this study and numerous other studies.^3,5,10^ Ochani et al.^17^ explain that micro thrombus formation is caused by the inflammation of lung tissue and pulmonary endothelial cells, which results in thrombotic complications such as pulmonary embolism, deep venous thrombosis (DVT), ischaemic stroke, myocardial injury and limb ischaemia. The pathogenesis of myocardial injury in patients with COVID-19 disease involves coronary spasm, hypoxic injury, microthrombi, hypercoagulability, hypoxic injury, direct vascular endothelial injury and atherosclerotic plaque instability, which increases the risk of acute myocardial infarction secondary to acute coronary occlusion.^13,14^

Almost all of our participants were prescribed antibiotics on admission (ceftriaxone with/without azithromycin). Antibiotics were prescribed in the institution as per the South African Standard Treatment Guidelines (STGs) and Essential Medicines List (EML). Without a point-of-care confirmation of the diagnosis, admitted patients were treated for community-acquired pneumonia based on their severity assessment as per the STGs. Antibiotics were stopped once the COVID-19 antigen or PCR tests confirmed COVID-19 infection.

Most of our participants required oxygen supplementation on admission. The decision to start HFNO_2_ and CPAP was based on the Horowitz index^9^ and the clinical picture of the patient while using less advanced oxygen modalities, that is, whether they were able to maintain arterial oxygen saturations above 88% when a 100% non-rebreather mask was used at 15–20 litres per min. High-flow nasal oxygen and CPAP machines were commonly required modalities that were unavailable during the first wave. A few HFNO_2_ and CPAP machines were acquired through private funding after the first wave of the COVID-19 pandemic. However, not every patient had the opportunity to go onto a device because of these resource constraints. Furthermore, many junior healthcare workers at the facility were untrained and inexperienced in using these devices, leading to poor utilisation of these devices and poor-quality management of patients on these devices. It is also possible that many patients who went on the HFNO_2_ survived and are not included in this mortality study.

Most participants requiring such modalities were discussed with the tertiary-level referral hospital; however, because of limited ICU beds and ventilators, many of our participants were put on waiting lists and were not transferred to higher levels of care. Discussion with the tertiary referral facility was reserved for patients who fulfilled the criteria for ICU admission and for those that required invasive ventilation. This decision to discuss with tertiary care was made by senior members of staff – usually family medicine consultants – within 24 h of admission. The reality of the resource-limited situation at the regional and tertiary hospitals was also well known, and many patients were not discussed, as clinicians in the district-level hospitals saw this as pointless in the current circumstances. Many patients also had morbid obesity, an exclusion criterion for the ICU. The clinical probability score was used to assist with the clinical diagnosis and not to determine the prognosis. However, the clinical frailty score (included in the admission clerking notes) assisted clinicians in deciding who to discuss for ICU care: patients with higher clinical frailty scores (greater than five) were not considered for ICU. The median length of admission to WWH before demise was 4 days, similar to figures found at a tertiary facility in the Western Cape.^2^

A meta-analysis across multiple electronic databases noted that case fatality rates (CFR) (COVID-19 deaths/COVID-19 PCR tests) varied depending on the population at stake.^23^ They found an overall pooled CFR of 10%, with a CFR of 13% in hospitalised patients, 37% in patients admitted to an ICU, 19% in patients older than 50 and only 1% in the general population. Our institutional crude fatality rate (institutional COVID-19 deaths/institutional COVID-19 admissions) of 15.3% is slightly higher than the crude fatality ratio found in hospitalised patients in this meta-analysis. This could be accounted for because of the limitations of a district hospital in dealing with severely ill patients because of inadequate critical care facilities, healthcare workers, level of expertise at this level and inadequate medical equipment.^24^ A study by Li et al.^25^ identified a negative correlation between the mortality rate of COVID-19 and test numbers per 100 people, government effectiveness score and the number of hospital beds. Thus, the increased crude fatality ratio found in our study may be highlighting the strain on the healthcare system during this period. A limitation of our study was our inability to accurately describe a case fatality ratio based on the fact that patients may have had multiple PCR tests over the defined period and may have presented with PCR results from other facilities, falsely impacting the case fatality ratio. The crude fatality ratio is therefore a relatively accurate way of determining the mortality rate of patients hospitalised with COVID-19 at WWH over specified time periods. Wave two (as characterised by the ‘Beta’ variant – 501Y.V2 or B.1.351) had the highest crude fatality ratio, indicating increased virulence of the variant.^26^ This is in keeping with the findings from multiple studies stating that the ‘Beta’ variant is more virulent than other COVID-19 strains because of its development of multiple protein mutations, causing increased infectivity of the strain and decreased neutralisation by existing vaccines.^27^ Eleven participants were dead on presentation to WWF. This may be because of late presentation, stigma and fear surrounding COVID-19 and healthcare facilities. Twenty-nine participants demised in the EC within a few hours of presentation, indicating the severity of the disease on presentation to the healthcare facility, 423 and implying late presentation.

Strengths of the study include the provision of a thorough description of the risk factors for mortality in a generalisable health context: an overwhelmed, resource-constrained DH in an LMIC. This provides representative data that can be used to provide insights into risk factors for COVID-19 mortality as well as managements in a similar health setting. Apart from those already mentioned, limitations of the study include some paucity of data because of scanty record-keeping and a reliance on paper-based clinical notes. This may have resulted in under-reporting of certain risk factors. Furthermore, there is a risk of false-positive COVID-19 cases because of the clinical probability score being used for participants without a positive COVID-19 PCR test.

## Conclusion

This study describes the COVID-19-related deaths during the first two years of the COVID-19 pandemic at a DH in South Africa. Many participants were female (60.1%), older than 60 (66.5%), of black African descent (59.6%), unvaccinated and had uncontrolled comorbidities. The institutional crude fatality rate was 15.3%, a value higher than that seen in many other hospitalised populations worldwide.^23^ This is perhaps indicative of the strain placed on an under-resourced facility with a lack of specialist medical expertise and a lack of advanced medical equipment. The highest crude fatality rate was seen in wave two (33.0%), which was characterised by the ‘Beta’ variant, perhaps indicating increased virulence of this variant.

Recommendations for future research include the development of an electronic record-keeping system, to allow the capture of reliable, site-specific information that could be used for quality improvement projects and further research as well as doing further research into the impact of the National Vaccination Programme on country-wide mortality statistics.
